# Reproducibility of a new device for robotic‐assisted TKA surgery

**DOI:** 10.1002/jeo2.70153

**Published:** 2025-02-18

**Authors:** Domenico Alesi, Vito Gaetano Rinaldi, Tosca Cerasoli, Davide Valente, Giulio Maria Marcheggiani Muccioli, Stefano Zaffagnini

**Affiliations:** ^1^ II Clinica Ortopedica e Traumatologica, IRCCS Istituto Ortopedico Rizzoli Bologna Italy

**Keywords:** Robin system, robotic‐assisted TKA, robotic surgery, TKA

## Abstract

**Purpose:**

Enhancing implant placement to achieve optimal gap balance is crucial in total knee arthroplasty (TKA). Given the limited precision of traditional instrumentation, tools like computer‐assisted surgery and robotic‐assisted TKA have emerged. This experimental cadaveric study aimed to evaluate the accuracy and reproducibility of the collaborative image‐free Robin robotic system to support its future clinical application.

**Methods:**

Fifteen cadaveric specimens were treated by eight experienced TKA surgeons. All surgeons, experts in computer‐assisted TKA but new to the Robin system, received standardized training. The Robin system uses a robotic arm to position and hold a universal cutting jig, while surgeons perform osteotomies. The indicator for registration repeatability was the alignment of the cutting block position with the previous pin placement. Bony resection, angles and axes were evaluated by comparing the preoperative planning values to the ones obtained with the Robin system with a validated navigation system.

**Results:**

There were no statistically significant differences between the planned and measured values for most resection angles, except for femoral and tibial orientation on sagittal plane (0.6 ± 0.8° and 0.6 ± 1.0°, respectively). Similarly, no statistically significant differences were recorded for resection thickness values, except for the distal medial femoral cut (0.8 ± 0.7 mm). Moreover, these results showed consistency among the different first‐time users.

**Conclusions:**

The study found that the Robin robotic system closely matched the preoperative plan for TKA, demonstrating high accuracy and consistency among first‐time users. This allows surgeons to easily achieve their planned targets without having to adapt their surgical technique, potentially improving both efficiency and outcomes even when handling complex cases.

**Level of Evidence:**

Not applicable.

**Abbreviations**
CT: computed tomography
TKA: total knee arthroplasty

## INTRODUCTION

Enhancing the placement of implants and achieving optimal gap balance are crucial goals in total knee arthroplasty (TKA). Traditional instrumentation has demonstrated limited precision, with up to 40% of cases deviating from the intended outcome [8, 10, 18]. While many surgeons continue to pursue the restoration of precise neutral limb alignment, alternative concepts have emerged, advocating for the preservation of a more anatomical alignment [19]. These methods aim to reduce the necessity for extensive ligament releases [4, 19].

Given the limitations of standard mechanical instrumentation, tools like computer‐assisted surgery, accelerometer‐based cutting jigs and robotic‐assisted TKA have emerged [1, 2, 13, 14]. The technological progress over the past two decades facilitated the evolution of robotic‐assisted surgery. Preoperative computed tomography (CT) scans and integration of various end effectors ranging from burrs to haptic saws, each with potential advantages and drawbacks [2, 14], significantly contributed to the development of robotic‐assisted TKA.

The most recently introduced robotic system even incorporates robotic placement of cutting jigs and dynamic ligament balance assessment. In this collaborative robotic system, a robotic arm handles and positions the cutting blocks, while the surgeon retains full control over sawing through the jig.

The collaborative Robin robotic system for TKA (Robin, Orthokey Italia) was developed with these features. Additionally, this system eliminates the need for preoperative imaging, expediting the intraoperative registration process, which can be customized according to surgeons' preferences. Despite the advantages of this approach, it should be noted that its precision is mostly dependent on the accuracy of landmarks registration performed by the users, which can have a significant effect on the alignment of the implant [5, 6, 16].

This experimental cadaveric study aimed to evaluate the accuracy of the Robin robotic system, and the reproducibility of landmarks registration between different users, in order to provide experimental support for its clinical application in the future. The primary end point is to evaluate the accuracy in bone resections, the secondary end point is to evaluate the efficacy of landmarks registration in terms of surgical time and reproducibility between different users.

## MATERIALS AND METHODS

In this study, 15 cadaveric specimens were used. Eight experienced TKA surgeons were involved, each operating on three or four specimens. Each specimen was treated by a couple of surgeons, to evaluate inter‐operator repeatability in landmarks acquisition and cutting block positioning.

Surgeons were experts in computer‐assisted TKA surgery (either navigated or robotic) but had no experience with this specific system. All surgeons had a minimum of 5 years of practice and a yearly caseload of over 100 knee arthroplasties. All surgeons received standardized training for the Robin system, including theoretical instruction on the system's features and hands‐on practice on sawbones. Each surgeon was free to choose the TKA implant to mitigate the bias associated with familiarity with a particular implant. The specific choice of implant was not significant because the robotic system is an open platform.

**Figure 1:**
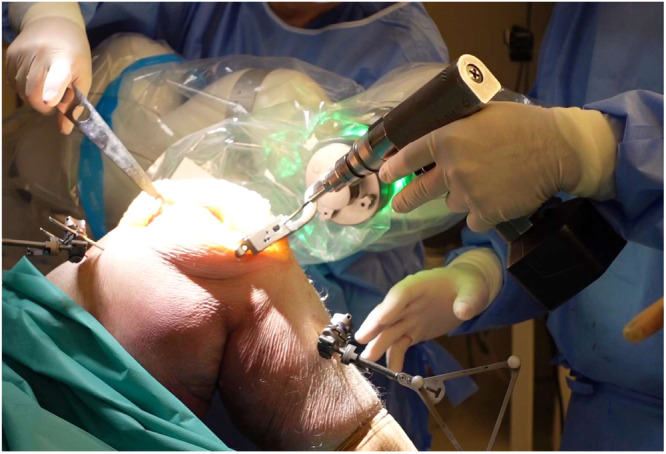
Pin insertion for jig fixation to the tibia before resections. The robotic arm holds the jig on the resection plane adjusting for knee movements.

The principle of Robin's Knee surgery is simple: the robotic arm positions and holds the universal cutting jig. The surgeon can move the cutting jig in the surgical area while the robot keeps it, in collaborative mode, on the resection plane. Once the cutting jig is set in the correct position, it is pinned to the bone by the surgeon, who then performs the osteotomies (Figure 1). Ligament balance can be performed before any resection and verified after each resection. Surgeon can verify medial and lateral gaps in extension and flexion. The aim of ligament balance phase is to minimize recutting and plan correct resections before execution. There is no preferred method for ligament balance, it can be ligament release or adjustment resection level. Since the Robin system can work with different implant designs, the correct position of the 4‐in‐1 cutting block is ensured by the robotic‐assisted execution of pin holes for the block.

### Robotic workflow

**Figure 2:**
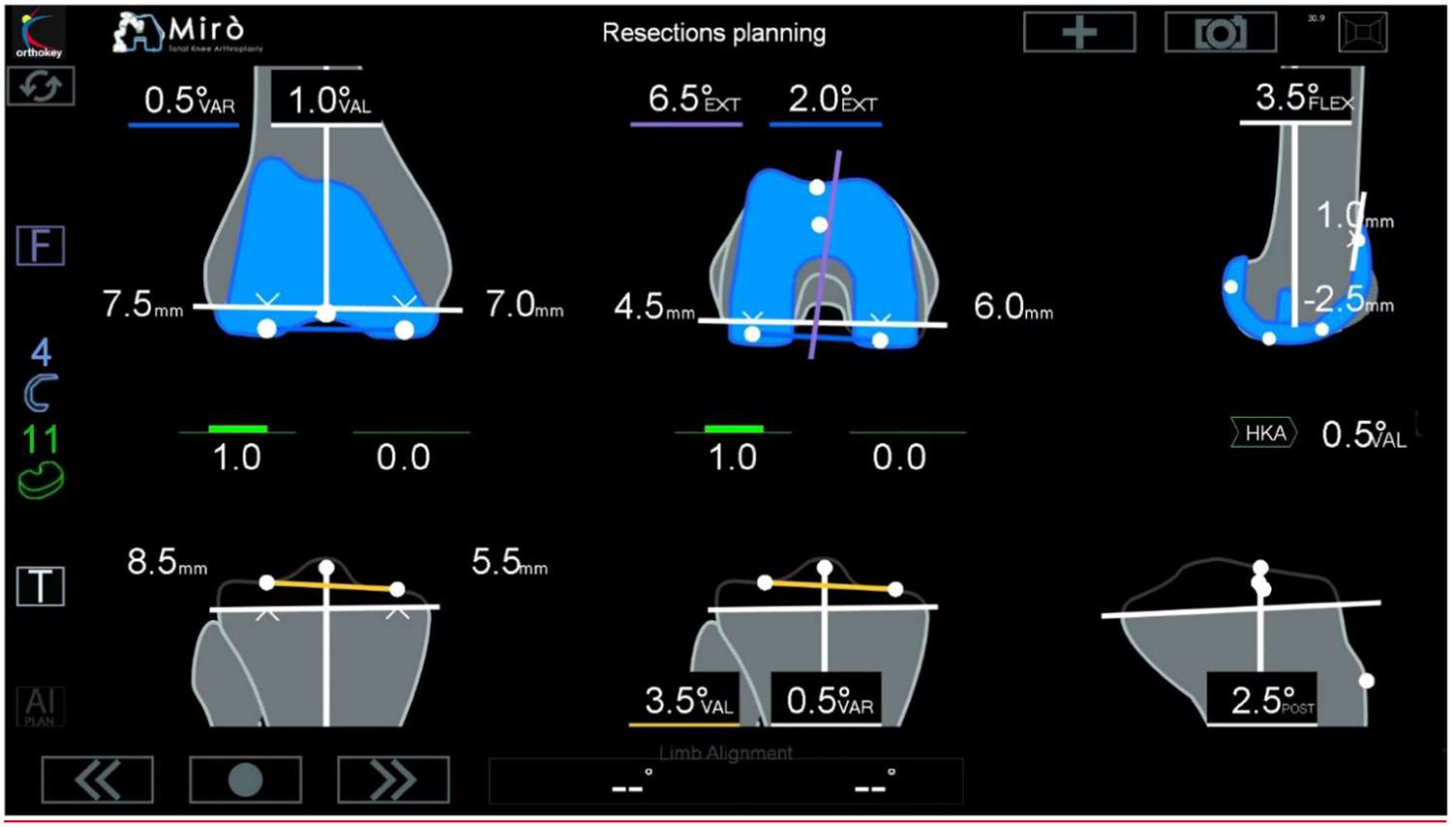
Screen interface during planning phase showing resection thickness, limb alignment and residual gap laxity according to implant positioning.

Two surgeons operated on the same specimen. Before accessing each cadaveric knee, the robot underwent calibration. Following surgical access, two rigid body trackers—one on the femur and one on the tibia—were implanted, and the registration of femoral and tibial landmarks was conducted. Subsequently, intraoperative planning was undertaken by each couple of surgeons at their discretion, utilizing dedicated software, to establish the optimal resection thickness and angle for achieving a well‐aligned and balanced TKA with the lowest polyethylene thickness (Figure 2). Cutting blocks for tibial and distal femoral resections were then positioned by the robotic arm, and pins for stabilization were placed. The cutting blocks were then removed, without performing osteotomies, while pins were kept in place.

In order to assess the inter‐operator repeatability of all procedures, after this step, the robotic‐assisted surgical procedure was restarted, and the second operator repeated the imageless registration process from the beginning. Then, the same surgical plan was applied. The cutting blocks were positioned by the surgeon while it was kept on the resection plane by the robotic arm. We checked if the user was able to position the cutting jig in front of previously placed pins and approach the bone surface, sliding pins into the block without forcing. Since the pins have a diameter of 3.2 mm, this can be considered as an inter‐operator repeatability of less than 2 mm (i.e., the centre of the pin is outside the hole radius on the cutting block).

The surgeons then performed the osteotomies. The thickness of each resected bone piece was measured by two independent observers using a calliper and compared to the planned resection values. The target angles from the intraoperative plan were assessed and compared with the bone cuts executed with the robotic system, evaluated using a validated computer‐assisted navigation system (BLU‐IGS, Orthokey Italia), acknowledged as the gold standard as per previous publications (Marcheggiani Muccioli GM F. S., 2021) [3, 7]. Afterwards, the surgeons proceeded with the placement of the chosen prosthetic implant.

Each surgeon could choose the preferred timing for assessing ligament balance in extension and flexion (at 0° and 90°). Ten out of 15 surgeons employed a tibial‐first approach, where tibial resection was planned and executed, ligament balance was assessed, and subsequently, femoral implantation was planned to achieve the desired leg alignment and ligament balance. Five surgeons opted for a distal femur‐first technique, where the distal femoral and tibial cuts were planned and executed to obtain the desired lower limb alignment. The flexion gap was always recorded to ensure adequate flexion stability, followed by the execution of the 4‐in‐1 resection. All planned values, including angles during all ranges of knee flexion and resection thickness, were documented for comparison with the operative plan.

According to the robotic surgical technique, cutting blocks are positioned by the robotic arm, while the surgeon keeps the ‘tactile feel’ by placing the pins for stabilization (as shown in Figure 1) and sawing through the jig. The robotic arm facilitates the positioning of the universal cutting jig according to the preoperative planning. However, the surgeon maintains full control over the bone cuts, as they perform the sawing manually.

### Navigation and angle measurement

In this investigation, osteotomies were executed according to the cutting block positioning of the robotic arm. The average accuracy of this infrared camera‐based imageless navigation system has been documented to be within 0.5 mm for resection thickness and 0.5° for angle measurement [3]. Identical rigid bodies were employed for both the Robin procedure and navigation measurements. During navigation measurements, the standard registration process coincided with the Robin bone registration procedure. For each osteotomy, a handheld validation tool was positioned on the cut to verify and document alignment in the sagittal and coronal planes. This dedicated tool is designed for measuring each bone cut, with a focus on the distal femoral cut and the tibial cut. The tool features a flat surface designed to align precisely with the cut surface, allowing for accurate navigation tracking. This enables the calculation of the disparity between the planned cut (depth, frontal and sagittal angles) and the cut executed using the robotic system.

### Evaluation of surgical time for landmarks registration

The landmarks registration time was standardized and recorded for all the surgeons, whereas the surgical procedure and its duration were at the surgeon's discretion.

### Evaluation of bony resections

Bony resection was evaluated by comparing the preoperative resection planned with the robotic system to the resection obtained after the osteotomy using a validated navigation system (BLU‐IGS, Orthokey Italia). Any differences in the frontal and sagittal planes and thickness were recorded.

The distal femoral cut and the tibial cut were measured with a calliper and compared with planned resection. To reduce bias between the planned resection and the caliper measurement the sawblade thickness of 1.5 mm was taken into account.

### Statistical analysis

Using the validated method of the navigation system [3], which acquired all the significant values throughout the entire procedure, was assessed the ability of the Robin robotic system to adhere to the preoperative plan. To verify data normality, descriptive statistics (mean, standard deviation and range) were computed. A dedicated *t* test for paired samples was employed to compare differences between target values and measured values, with the navigation system and the caliper regarded as the gold standard. The proportion of differences falling within ±1° and ±2° was determined for alignment values, while the proportion within ±1 and ±2 mm was calculated for resection thicknesses. Prediction intervals for a single future measurement were also computed to delineate the range where planned and measured values would likely converge. Prior to analysis, confidence intervals of 95% were predefined for both the *t* test and the 95% prediction interval. Statistical significance was established at *p* < 0.05 for all statistical assessments. Analyse‐it v2.30 for Microsoft Excel (Analyse‐it Software, Ltd) was utilized for all statistical computations. Sample size has been defined in order to have a power, in detecting differences >1 mm, >0.8.

## RESULTS

### Accuracy of osteotomies

For all 15 cases, the difference between planned and executed osteotomies followed a normal distribution. Tables 1 and 2 show the comparison between planned and executed resections. Difference in orientation (Table 1) on frontal and sagittal planes, compared to previously validated navigation method, shows an average difference <1° with no values >2°. Same results can be observed when comparing planned resection with caliper measured osteotomies (Table 2).

**Table 1:** Mean difference in orientation between planned resection with robotic system and verified resection with navigation system on distal femoral and tibial cut.

Rotation	Mean ± st. dev (°)	Range (°)	Within 1°	Within 2°	95% CI
Fem frontal	0.2 ± 0.8	−1.5 to 1.5	93%	100%	−0.17 to 0.59
Fem sagittal*	−0.6 ± 0.8	−1.5 to 1.5	73%	100%	−1.09 to −0.13
Tib frontal	−0.3 ± 0.8	−2.0 to 1.5	73%	100%	−0.84 to 0.17
Tib sagittal*	−0.6 ± 1	−1.8 to 1.0	67%	100%	−1.02 to −0.18

Abbreviation: CI, confidence interval.

* *p* < 0.05.

**Table 2:** Mean difference between planned resection with robotic system and resection measured with calliper.

Osteotomy	Mean ± st. dev (mm)	Range (mm)	Within 1 mm	Within 2 mm	95% CI
Medial condyle*	0.8 ± 0.7	−0.4 to 1.9	67%	100%	0.41–1.11
Lateral condyle	0.6 ± 0.9	−1.0 to 1.8	60%	100%	0.11–1.06
Medial plateau	0.3 ± 0.6	−0.5 to 1.2	80%	100%	−0.04 to 0.54
Lateral plateau	0.6 ± 0.8	−1.0 to 1.8	60%	100%	0.22–1.02

Abbreviation: CI, confidence interval.

* *p* < 0.07.

### Registration

The time required for registration was 81 ± 14 s, ranging from 58 to 120 s. There was no statistical difference (*p* < 0.05) between the eight different surgeons. Cutting block repositioning by second operator over previously placed pins was always successful without forcing.

## DISCUSSION

The most important finding of the present study was that cuts performed using the Robin system closely matched the preoperative plan. There were no statistically significant differences between the planned and the measured values for most resection angles, except for femoral and tibial orientation on sagittal plane, which exhibited a mean difference of 0.6° from the planned one. Similarly, no statistically significant differences were recorded for resection thickness values, except for the distal medial femoral cut which was on average 0.8 mm and never more than 2 mm. This is particularly significant because all the users were at their first experience with the Robin robotic system and were fully within the learning curve typical of Robotic devices [11].

These results are in line with the current literature. The study by Paratte et al. involving surgeons with a learning curve of five cases, reports similarly promising results, with a sub‐millimetric average difference between planned and executed cuts with a distribution within 2 mm for >97% of cases [17]. While the potential inaccuracies of robotic devices in rotational alignment have already been reported in other studies, such as the one by Shin et al., which observed a reduction in sagittal rotation accuracy with a similar robotic device [21] during the first 20 cases, several studies conducted between 2022 and 2023 highlighted the ability of various robotic systems to reduce alignment outliers and improve accuracy compared to manual instruments in TKA [9, 12, 20, 22]. Despite the high accuracy of the robotic system, potential deviations between the planned and executed cuts could be due to variables related to the bone quality, such as sclerotic bone, or to the bending of the saw. Indeed, several authors have demonstrated that longer sawing distances, blade stiffness and cutting stability are critical factors for cutting accuracy [15].

The collaborative robotic systems described in this paper are image‐free and involve the robot positioning and holding the cutting jigs while the surgeon executes the cuts with a conventional saw. This design was chosen to facilitate a shorter setup time compared to other robotic devices. This is achieved through rapid and easy landmarks acquisition without bone morphing or extensive preoperative planning. The system is compatible with all implant models, and bone resections require only one cutting jig.

To highlight the efficiency of this robotic system in terms of registration time and cutting block positioning, only first‐time users were involved. Even for surgeons who had never used this system before, the registration process was completed in an average time of 81 s, with no statistical difference observed among different surgeons. The acquisition of the anatomical landmarks is crucial to achieve the required lower limb alignment and may lead to significant errors if done incorrectly. Moreover, the cutting block repositioning over previously placed pins was consistently successful without any need for forcing, indicating the reliability of the robotic system in the surgical workflow.

The novelty of this study, compared to previous published works is that it is the first cadaveric evaluation of a novel robotic device not yet commercially available. The working principle is similar to state‐of‐the‐art, and results were expected to be in line with what was already reported. In addition, most papers report the accuracy of robotic devices after an initial learning curve for multiple users or report the learning curve of just one centre. As far as we know, nobody reported the accuracy results of first‐time users, with no previous training. Finally, this is the first study analyzing the accuracy of an open‐platform robot. The advantage of such a system is that it does not bind the surgeon to the use of a single type of implant, leaving him free to choose the most suitable one according to the patient's characteristics.

Despite these promising results, several limitations should be acknowledged. First, this study was conducted on cadaveric specimens and the translation of these findings to patient populations requires further investigations. Additionally, the low sample size does not allow to generalize the obtained results. Finally, there is no strong evidence that this increased accuracy may lead to better clinical results.

## CONCLUSIONS

The study found that the Robin robotic system closely matched the preoperative plan for TKA, demonstrating high accuracy and consistency among first‐time users. This allows surgeons to easily and intuitively achieve their planned targets without having to adapt their surgical technique, potentially improving both efficiency and outcomes even when handling complex cases.

## AUTHOR CONTRIBUTIONS

Giulio Maria Marcheggiani Muccioli and Stefano Zaffagnini conceived and supervised the study. Davide Valente and Vito Gaetano Rinaldi contributed to data collection and analysis. Tosca Cerasoli and Domenico Alesi participated in manuscript drafting and review. All authors have read and approved the final version of the manuscript.

## CONFLICT OF INTEREST STATEMENT

The authors declare no conflicts of interest.

## ETHICS STATEMENT

As this is a cadaveric study, no ethics approval or consent was required.

## Data Availability

The data generated and analyzed during this study are available upon request from the Rizzoli Orthopaedic Institute (IOR).
